# Attenuated Late-Phase *Arc* Transcription in the Dentate Gyrus of Mice Lacking *Egr3*

**DOI:** 10.1155/2017/6063048

**Published:** 2017-05-15

**Authors:** Amanda Maple, Rachel E. Lackie, Diana I. Elizalde, Stephanie L. Grella, Chelsey C. Damphousse, Collin Xa, Amelia L. Gallitano, Diano F. Marrone

**Affiliations:** ^1^Department of Basic Medical Sciences, University of Arizona, Phoenix, AZ 85004, USA; ^2^Department of Psychology, Wilfrid Laurier University, Waterloo, ON, Canada N2L 3C5; ^3^McKnight Brain Institute, University of Arizona, Tucson, AZ 85724, USA

## Abstract

The dentate gyrus (DG) engages in sustained *Arc* transcription for at least 8 hours following behavioral induction, and this time course may be functionally coupled to the unique role of the DG in hippocampus-dependent learning and memory. The factors that regulate long-term DG *Arc* expression, however, remain poorly understood. Animals lacking *Egr3* show less *Arc* expression following convulsive stimulation, but the effect of *Egr3* ablation on behaviorally induced *Arc* remains unknown. To address this, *Egr3*^−/−^ and wild-type (WT) mice explored novel spatial environments and were sacrificed either immediately or after 5, 60, 240, or 480 minutes, and *Arc* expression was quantified by fluorescence in situ hybridization. Although short-term (i.e., within 60 min) *Arc* expression was equivalent across genotypes, DG *Arc* expression was selectively reduced at 240 and 480 minutes in mice lacking *Egr3*. These data demonstrate the involvement of *Egr3* in regulating the late protein-dependent phase of *Arc* expression in the DG.

## 1. Introduction

The hippocampus is well established as a brain structure critical to many forms of memory. As a result, a number of studies have investigated how hippocampal neurons modify synaptic connections to permit information storage and retrieval. These synaptic changes are the result of signaling cascades that include immediate early gene (IEG) expression [1]. One of these IEGs, activity-regulated cytoskeleton-associated protein (*Arc*, *Arg3.1*), is required for both long-term potentiation (LTP) and long-term depression in the hippocampus, as well as for lasting memory formation [2–5].

Within the dentate gyrus (DG) of the hippocampus, *Arc* differs from most other IEGs in that it can be expressed for up to 8 hours after initial induction [6, 7]. This sustained *Arc* expression is required for lasting LTP in DG granule cells and thus likely critical for the synaptic changes involved in forming long-term memories that depend on the DG [5, 8]. Importantly, DG *Arc* expression in response to spatial learning occurs in a sequential cascade. Immediately following behavioral induction, *Arc* is expressed predominantly in the dorsal suprapyramidal blade (DG_SP_) of the DG [9]. Elevated *Arc* also becomes apparent in the ventral infrapyramidal blade (DG_IP_) after approximately 4 hours [7], and *Arc* transcription continues in both blades for at least 8 hours following behavioral induction. Furthermore, DG *Arc* expression at these long delays is correlated with spatial memory performance [6]. These observations are consistent with the idea that sustained expression of behaviorally induced *Arc* in the DG is functionally coupled to the formation of stable spatial memories. However, the molecular mechanism that sustains *Arc* expression for hours following an environmental stimulus remains unknown.

As an IEG, *Arc* is rapidly activated in response to neuronal activity [10]. Unlike the initial stimulation of *Arc*, however, the late phase of DG *Arc* transcription (i.e., hours after induction) requires protein synthesis. The IEG transcription factor early growth response 3 (*Egr3*) is required for late-phase *Arc* transcription in DG in response to convulsive stimulation [11]. Importantly, *Egr3*^−/−^ mice do not lack the ability to transcribe *Arc*, as expression appears normal in these mice shortly after a pharmacologically induced seizure. However, sustained *Arc* expression is absent 4 hours after seizure onset. After exposure to a novel environment, *Egr3* is also activated in the DG in the same cells that express *Arc* [11]. These findings suggest that *Egr3* may also mediate enduring *Arc* transcription during behavior and thus play a pivotal role in both learning and plasticity as evinced by the deficits seen in animals lacking *Egr3* [11, 12]. However, sustained *Arc* expression in mice lacking *Egr3* has only been examined following supraphysiological levels of stimulation, and expression patterns of gene products in the DG produced by this type of robust stimulation can be different from those produced by behavior (e.g., [6, 13]).

To address this issue, we examined sustained *Arc* expression using fluorescence in situ hybridization (FISH) in the DG of *Egr3^−/−^* mice and their WT littermates immediately after a 5-minute exposure to a novel environment, as well as after 60-, 240-, and 480-minute delays. These data will establish how the time course of behaviorally induced *Arc* expression is altered in animals lacking *Egr3*.

## 2. Materials and Methods

### 2.1. Subjects

Previously generated male and female *Egr3*^−/−^ mice [14] were back-crossed to C57BL/6 mice for greater than 25 generations. Animals were housed on a 14/10 h light/dark schedule with ad libitum access to food and water. Studies were conducted on littermate *Egr3*^+/+^ and *Egr3*^−/−^ progeny of heterozygous breeding.

### 2.2. Behavioral Procedures

Animals explored a novel environment as previously described [6, 15]. Briefly, mice were divided into 5 behavioral groups (*n* = 6–9 mice/group/genotype) consisting of littermate-matched pairs of *Egr3*^−/−^ and WT mice. The control group remained undisturbed in their cages until sacrifice. The four experimental groups were exposed to a novel open-field environment (a 78 × 37 cm clear plastic enclosure). The open field was divided into 9 equal grids. Mice were permitted to explore the environment for 5 minutes. The exploration sessions of all mice were recorded using a webcam and tracked post hoc at Wilfrid Laurier University using Any-maze tracking software (Stoelting, Kiel, WI). At the end of 5 minutes, one group (0) was immediately sacrificed. Three groups were sacrificed after a delay period of 60, 240, or 480 minutes. Mice were returned to their cages in the animal colony for the duration of the postexposure interval. Sacrifice was performed by overdose with isoflurane followed by decapitation. Brains were rapidly removed and flash frozen in isopentane submerged in slurry of ethanol and dry ice. The open-field environments were thoroughly cleaned between subjects. The caged control group was sacrificed directly from the home cage as a negative control to establish baseline levels of *Arc* expression. The brains were then shipped to Wilfrid Laurier University on dry ice for in situ hybridization as previously described [6, 16].

### 2.3. In Situ Hybridization

**Figure 1:**
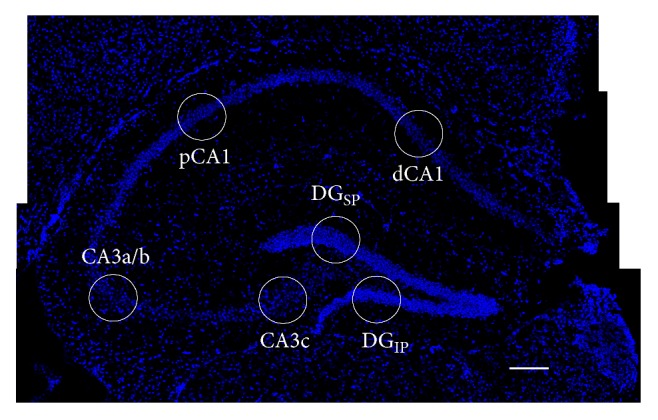
Hippocampal imaging. A sample montage of dorsal hippocampus labelled with DAPI (blue, scale bar = 100 *μ*) depicts representative imaging locations (circles) for the regions described in this study in both pyramidal cells (distal (dCA1) and proximal (pCA1) CA1, CA3a/b, and CA3c) and granule cells (suprapyramidal (DG_SP_) and infrapyramidal (DG_IP_) dentate gyrus).

The frozen brains were embedded in optimal cutting temperature (OCT) medium (Fischer Scientific, Whitby, ON) in blocks that included tissue from every behavioral group. Coronal sections (20 *μ*m thick) were obtained from each block and collected on slides treated with 3-aminopropyltriethoxysilane (Sigma-Aldrich, Oakville, ON) and stored at −80°C. Once thawed, sections were hybridized for 18 hours with full-length *Arc* riboprobes generated using MAXIscript kits (Ambion, Austin, TX) and digoxigenin-UTP labelling mix (Roche, Indianapolis, IN). Slides were subsequently incubated with anti-digoxigenin-peroxidase (1 : 400; Roche) for 2 h at RT, followed by Cy3 for 30 min at RT (1 : 50; PerkinElmer, Waltham, MA) and DAPI for 30 min at RT (Sigma-Aldrich) to stain nuclei. An Olympus FV1000 laser scanning confocal microscope obtained z-stacks (20 *μ*m thickness, ~1.0 *μ*m optical planes, and 0.7 *μ*m interval between planes) in one random location in the DG_IP_ and two locations in the DG_SP_, as well as from one random location in each of the proximal and distal regions of CA1, CA3a, and CA3c (see Figure 1). Neurons within the median of 20% of each z-stack were classified as *Arc+* or *Arc−*. The total number of cells counted in each region is provided in Table 1.

### 2.4. Statistical Analyses

Statistical analyses consisted of either one- or two-way analysis of variance (ANOVA) followed by post hoc analyses using Tukey's HSD.

## 3. Results

### 3.1. *Egr3* Does Not Significantly Alter Exploration Behavior in a Novel Space

Because *Egr3^−/−^* mice demonstrate both motor deficits [14] and hyperactivity [12], the locomotor behavior of all mice was analyzed. An ANOVA on the mean path length travelled by *Egr3^−/−^* (10.94 ± 2.31 m) and WT (13.73 ± 1.80 m) mice showed no significant difference (*F*_1,78_ = 1.72; *p* = 0.19).

### 3.2. *Egr3* Does Not Significantly Alter *Arc* Expression in Hippocampal Pyramidal Cells

**Figure 2:**
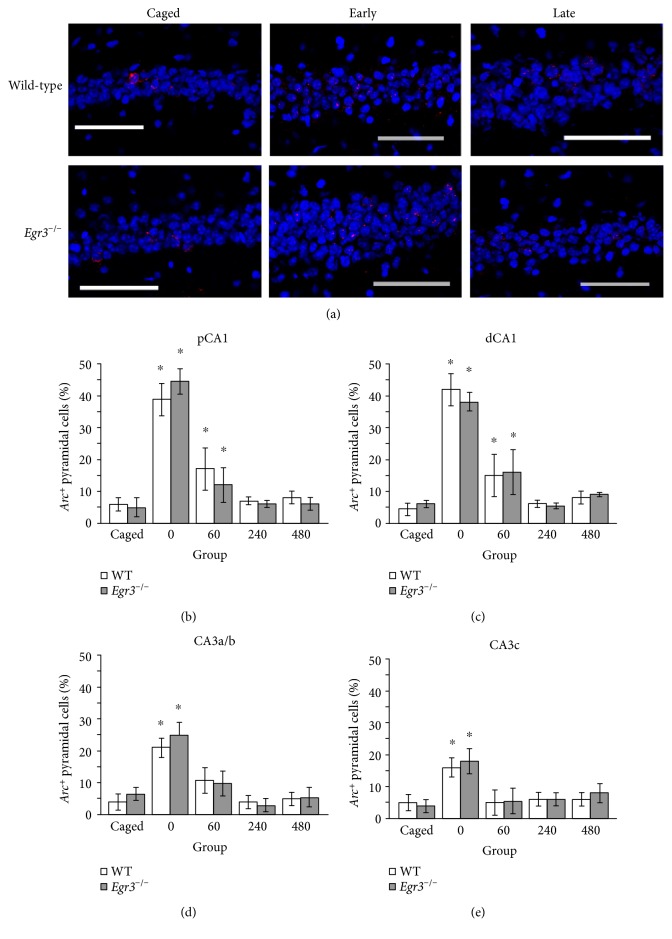
Normal exploration-induced *Arc* expression in pyramidal cells in *Egr3*^−/−^ mice. Sample confocal images are provided (a) of proximal CA1 from WT (top row) and *Egr3*^−/−^ (bottom row) mice under baseline control (caged) conditions, versus 0 minutes (early) and 480 minutes (late) following a five-minute exposure to a novel environment (red = *Arc*, blue = DAPI, scale bar = 100 *μ*m). Consistent with these images, graphs of (b) proximal CA1, (c) distal CA1, (d) CA3a/b, and (e) CA3c all show that novel exploration induces a robust increase in *Arc* expression immediately (0′) that is greatly reduced by 60′. At 240′ and 480′, *Arc* expression is no different from caged controls in any pyramidal cell region. No differences are observed between *Egr3*^−/−^ and WT mice (^∗^*p* < 0.05 relative to caged control of the same genotype; graphs display mean ± SEM).

Figure 2(a) shows representative images of the results of *Arc* FISH in CA1 from *Egr3*^−/−^ and WT mice at baseline and at two time points (during the early, protein synthesis-independent phase of *Arc* transcription, as well as during the late, protein synthesis-dependent phase of *Arc* transcription) following behavioral exposure to a novel environment. The transcription of *Arc* is observed in very few cells in mice that remain undisturbed in their home cages (baseline cage control). Five minutes of spatial exploration induces a robust increase in *Arc* expression in both WT and *Egr3^−/−^* mice that can be observed immediately, and that is absent at 480 minutes.

A two (genotype) by four (region: proximal CA1, distal CA1, CA3a/b, and CA3c) by five (behavioral group) ANOVA revealed a main effect of both behavioral group (*F*_4,156_ = 58.33; *p* < 0.001) and region (*F*_4,156_ = 47.84; *p* < 0.001), as well as a behavioral group by region interaction (*F*_4,156_ = 14.78; *p* < 0.001). These effects indicate that, although *Arc* expression was robustly increased in all pyramidal cell regions by brief spatial experience, more cells expressed *Arc* in the CA1 regions relative to CA3, consistent with previous literature [16–18]. No main effect of genotype (*F*_1,156_ = 2.07; *p* = 0.16) or genotype by behavioral group interaction (*F*_4,156_ = 1.56; *p* = 0.25) was observed. Thus, *Arc* expression remains normal in the pyramidal cells of *Egr3*^−/−^ mice.

### 3.3. *Egr3* Selectively Regulates Late Phase of *Arc* Transcription in DG Granule Cells

**Figure 3:**
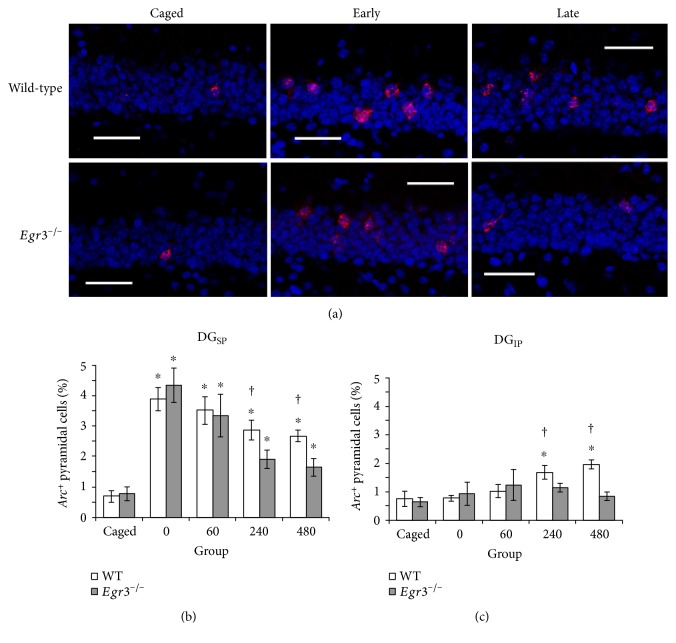
Accelerated loss of exploration-induced *Arc* expression in the DG of *Egr3*^−/−^ mice. Sample confocal images are provided (a) of the DG suprapyramidal blade (DG_SP_) from WT (top row) and *Egr3*^−/−^ (bottom row) mice under baseline control (caged) conditions, versus 60 minutes (early) and 480 minutes (late) following a five-minute exposure to a novel environment (red = *Arc*, blue = DAPI, scale bar = 100 *μ*m). Consistent with these images, graphs of DG_SP_ (b) show that novel exploration induces *Arc* expression immediately (0′) in all mice and that in WT mice *Arc* transcription persists throughout all time points observed. In *Egr3^−/−^* mice, however, exploration-induced *Arc* expression is selectively reduced in the late, protein synthesis-dependent phase of transcription (i.e., 240′ and 480′) relative to their WT littermates. In contrast, significant induction of *Arc* expression in the DG infrapyramidal blade (b) is not apparent in WT mice until 240 min postexploration and is completely absent in *Egr3*^−/−^ mice (^∗^*p* < 0.05 relative to caged control of the same genotype; ^†^*p* < 0.05 relative to *Egr3^−/−^*; graphs display mean ± SEM).

Figure 3(a) shows representative images of the results of *Arc* FISH in the DG suprapyramidal blade from *Egr3*^−/−^ and WT mice at baseline and at two time points (one during the early, protein synthesis-independent phase of *Arc* transcription and one during the late, protein synthesis-dependent phase of *Arc* transcription) following behavioral exposure to a novel environment. Transcription of *Arc* is observed in very few cells in mice that remain undisturbed in their home cages (baseline cage control). Five minutes of exploration of a novel environment induces a robust increase in *Arc* expression in both WT and *Egr3^−/−^* mice within 60 minutes. By 480 minutes following the novel exploration, however, *Arc* mRNA levels decreased substantially in *Egr3^−/−^* mice compared to their WT littermates.

Figure 3(b) illustrates that, in the DG_SP_, *Arc* expression is induced to the same level in *Egr3^−/−^* mice at 5 min and 60 min. As previously reported [6, 7, 9, 19], this brief exposure results in an increase in the number of granule cells expressing *Arc* in WT mice that perdures for at least 8 hours. However, by 240 min after exposure, the levels of *Arc* are reduced significantly more in the *Egr3*^−/−^ mice than their WT littermates, and this effect persists at 480 min. In the DG_IP_, the percentage of *Arc*-expressing granule cells was increased in WT mice at 240 and 480 min following exploration, while no significant increase was seen in *Egr3*^−/−^ mice (Figure 3(c)).

A two (genotype) by two (region: DG_SP_ versus DG_IP_) by five (behavioral group) ANOVA revealed a main effect of both behavioral group (*F*_4,138_ = 16.75; *p* < 0.001) and region (*F*_4,138_ = 64.23; *p* < 0.001), as well as a behavioral group by region interaction (*F*_4,138_ = 9.27; *p* < 0.001). These effects indicate that the blades of the DG show unique responses to brief spatial experience, consistent with previous literature [6, 7, 9, 19]. Although no main effect of genotype was observed (*F*_1,138_ = 0.13; *p* = 0.72), a significant genotype by behavioral group interaction (*F*_4,138_ = 6.29; *p* < 0.0001) was observed. This interaction demonstrates that while *Arc* expression remains normal in *Egr3*^−/−^ mice both during rest (i.e., in caged controls) and early after behavioral induction (i.e., in the 5′ and 60′ groups), the late phase of *Arc* expression (i.e., 240′ and 480′) is significantly reduced in mice lacking *Egr3*.

## 4. Discussion

The results reported here demonstrate that *Egr3* selectively regulates the DG-specific perdurance of *Arc* transcription. In the pyramidal cell fields of the hippocampus, *Arc* expression was not changed in mice lacking *Egr3*. In contrast, the DG of *Egr3*^−/−^ mice demonstrated a robust and selective knockdown in the late, protein synthesis-dependent phase of *Arc* transcription several hours after behavioral induction. Moreover, the blunting of the *Arc* transcriptional response to spatial processing was particularly pronounced in the DG_IP_. In WT animals, this blade only expressed more *Arc* than caged controls at 4 and 8 hours after behavior, and this response was absent in *Egr3*^−/−^ mice. These findings reaffirm the importance of validating observations made following convulsive stimulation by investigating conditions of behavioral induction, since the time course of *Arc* expression in the DG_SP_ and DG_IP_ we observed in response to physiological conditions has not been reported in response to seizure. In the DG_SP_, the knockdown of behaviorally induced *Arc* observed is considerably more subtle (an approximately 50% knockdown, rather than a compete ablation) than that which has been described following supraphysiological stimulation [11]. This apparent discrepancy, however, may in fact be the result of methodology. Unlike previous work, which utilized semiquantitative analysis of precipitate-labelled PCR products, the current study employed single-cell analysis of fluorescence-conjugated, full-length riboprobes made by reverse transcription. Collectively, these changes result in an order of magnitude greater sensitivity in the current data, and thus greater probability of detecting low abundance *Arc* expression in the DG of *Egr3*^−/−^ mice. This more moderate knockdown of *Arc* expression, however, still carries the potential to profoundly impact memory.

Our understanding of the functional role of sustained *Arc* transcription in the DG in supporting memory remains in its infancy. Both experimental evidence [20] and computational models [21] suggest that DG granule cells play a role in “tagging” the relative timing of events. It has been proposed that sustained *Arc* expression may provide a molecular mechanism to keep representations labile within the DG to sculpt and ultimately disambiguate representations for events that happen on the scale of hours [19]. Testing these ideas will be greatly facilitated by the identification of mechanisms that uniquely drive late-phase *Arc* transcription so that it may be selectively manipulated. The current data indicate that *Egr3* is a viable target for such a manipulation to decipher the specific contribution of late-phase *Arc* transcription to hippocampus-dependent learning and memory in intact animals.

## Figures and Tables

**Table 1:** Mean number of cells per animal counted across the hippocampal formation.

Genotype	Group	Region					
		Proximal CA1^1^	Distal CA1^1^	CA3a^2^	CA3c^2^	DG_SP_	DG_IP_
*WT*	*Caged*	297.4 ± 18.3	353.8 ± 11.2	168.5 ± 15.6	224.6 ± 20.8	1782.5 ± 382.7	1424.3 ± 269.3
	*0*	330.8 ± 14.3	337. 6 ± 12.1	202.5 ± 9.2	157.0 ± 9.6	1620.6 ± 330.5	1213.9 ± 386.7
	*60*	305.7 ± 20.2	285.4 ± 15.2	189.1 ± 8.7	212.0 ± 18.4	1662.5 ± 263.3	1487.5 ± 283.2
	*240*	263.1 ± 13.3	335.1 ± 9.6	154.5 ± 11.1	294.0 ± 12.1	1726.7 ± 280.9	1322.2 ± 279.1
	*480*	305.4 ± 11.4	319.0 ± 19.1	209.8 ± 9.0	252.8 ± 15.0	1905.2 ± 324.9	1610.8 ± 253.2
*Egr3^−/−^*	*Caged*	360.8 ± 14.9	309.6 ± 19.0	135.5 ± 5.4	189.9 ± 13.6	1980.2 ± 324.9	1380.1 ± 254.8
	*0*	348.2 ± 23.1	297.3 ± 18.1	180.8 ± 15.8	150.7 ± 9.3	1816.9 ± 317.4	1554.7 ± 262.6
	*60*	315.3 ± 16.3	347.0 ± 18.6	101.5 ± 18.0	240.8 ± 16.8	1722.1 ± 296.8	1442.5 ± 256.5
	*240*	415.1 ± 21.8	326.3 ± 17.8	216.8 ± 19.8	217.2 ± 9.0	1835.6 ± 309.1	1322.2 ± 234.6
	*480*	227.9 ± 27.7	354.9 ± 14.0	175.4 ± 7.4	194.0 ± 9.4	2015.6 ± 314.3	1493.3 ± 261.9

^1^In a transverse section of the hippocampus, proximal CA1 is the region closest to CA2, while distal CA1 is closest to the subiculum (see Figure 1).

^2^In a transverse section of the hippocampus, CA3a is the region closest to CA2, while CA3c is closest to the DG (see Figure 1).
